# Animal-Borne Imaging Reveals Novel Insights into the Foraging Behaviors and Diel Activity of a Large-Bodied Apex Predator, the American Alligator (*Alligator mississippiensis*)

**DOI:** 10.1371/journal.pone.0083953

**Published:** 2014-01-15

**Authors:** James C. Nifong, Rachel L. Nifong, Brian R. Silliman, Russell H. Lowers, Louis J. Guillette, Jake M. Ferguson, Matthew Welsh, Kyler Abernathy, Greg Marshall

**Affiliations:** 1 Department of Biology, University of Florida, Gainesville, Florida, United States of America; 2 School of Natural Resources and Environment, University of Florida, Gainesville, Florida, United States of America; 3 InoMedic Health Applications, Merritt Island, Florida, United States of America; 4 Department of Obstetrics and Gynecology and Hollings Marine Laboratory, Medical University of Charleston South Carolina, Charleston, South Carolina, United States of America; 5 Guana Tolomato Matanzas National Estuarine Research Reserve, Marineland, Florida, United States of America; 6 National Geographic Remote Imaging, Washington, District of Columbia, United States of America; Texas A&M University, United States of America

## Abstract

Large-bodied, top- and apex predators (e.g., crocodilians, sharks, wolves, killer whales) can exert strong top-down effects within ecological communities through their interactions with prey. Due to inherent difficulties while studying the behavior of these often dangerous predatory species, relatively little is known regarding their feeding behaviors and activity patterns, information that is essential to understanding their role in regulating food web dynamics and ecological processes. Here we use animal-borne imaging systems (Crittercam) to study the foraging behavior and activity patterns of a cryptic, large-bodied predator, the American alligator (*Alligator mississippiensis*) in two estuaries of coastal Florida, USA. Using retrieved video data we examine the variation in foraging behaviors and activity patterns due to abiotic factors. We found the frequency of prey-attacks (mean = 0.49 prey attacks/hour) as well as the probability of prey-capture success (mean = 0.52 per attack) were significantly affected by time of day. Alligators attempted to capture prey most frequently during the night. Probability of prey-capture success per attack was highest during morning hours and sequentially lower during day, night, and sunset, respectively. Position in the water column also significantly affected prey-capture success, as individuals’ experienced two-fold greater success when attacking prey while submerged. These estimates are the first for wild adult American alligators and one of the few examples for any crocodilian species worldwide. More broadly, these results reveal that our understandings of crocodilian foraging behaviors are biased due to previous studies containing limited observations of cryptic and nocturnal foraging interactions. Our results can be used to inform greater understanding regarding the top-down effects of American alligators in estuarine food webs. Additionally, our results highlight the importance and power of using animal-borne imaging when studying the behavior of elusive large-bodied, apex predators, as it provides critical insights into their trophic and behavioral interactions.

## Introduction

Large-bodied, apex predators have the potential to shape communities through cascading effects of consumptive and non-consumptive interactions with lower trophic levels [1]. Historically, studies relied on limited data from direct observation [2] and/or indirect estimates (e.g., allometry and metabolic scaling) to parameterize and inform ecological models (e.g., Ecosim, Ecopath) used to predict the potential impacts these species have on community and ecosystem processes [3], [4], [5]. While these techniques provide accurate estimates of minimum consumption rates necessary to sustain metabolic processes and allow for partial observations of animal behavior, they are limited in determining the frequency of feeding events, as well as the success of capture attempts, and do not allow for assessment of variation in these behaviors. Furthermore, the incorporation of species- and system-specific data into ecological models as well as behavioral interactions can provide more detailed predictions regarding the impacts of species interactions on communities and ecosystems [4], [6].

Over the past 30 years, animal-borne imaging and environmental data collection systems (AVEDs) have provided researchers an opportunity to gather animal point-of-view video and audio data in the absence of observers [7], [8]. Developed by the National Geographic Remote Imaging Program, Crittercam is an AVED system that has been used to study behavioral, physiological, and ecological interactions in more than 60 vertebrate and invertebrate species. Here, we use video data collected by Crittercam deployments to study the behavior and diel activity patterns of adult American alligators (*Alligator mississippiensis*, Daudin 1802) in two Atlantic estuaries in Florida.

While listed as an endangered species in 1967, subsequent management success and sustainable use has enabled American alligators to become highly abundant large-bodied apex predators in aquatic habitats throughout their native range in the United States’ southeastern coastal plain. Alligators inhabit a wide range of aquatic ecosystems from freshwater rivers, swamps, lakes, and marshes to brackish marshes, mangrove swamps, salt marshes, and estuaries [9], [10]. Furthermore, alligators are ecologically important to many aquatic systems. Their population status, reproductive success, and body condition can serve as indicators of ecosystem health [11]. Alligators provide refuge habitat and essential resources for both plant and animal species by constructing and maintaining nest mounds and shallow pools termed ‘alligator holes’ [12], [13], [14], [15]. The movement of alligators between habitats increases ecosystem connectivity and potentially mediates nutrient transfers between isolated ecosystems [16], [17]. Additionally, a number of studies provide evidence that alligators may play important roles in community- as well as ecosystem-level processes through their indirect effects within food webs [13], [18], [19], [20].

American alligator food habits have been extensively studied across many freshwater populations (i.e., lakes, freshwater marshes) using stomach content analyses (e.g., [21], [22]). Yet, other than a few anecdotal accounts of feeding behaviors (e.g., [23], [24], [25]), surprisingly little is known regarding the frequency of prey-capture attempts or rates of prey-capture success in natural environments, particularly in estuarine ecosystems.

As is the case with American alligators, information concerning crocodilian foraging behavior has been gathered from stomach content analyses (e.g., [22], [26], [27]); anecdotal observations of cooperative feeding, specialized feeding behaviors, and mass aggregation events (e.g., [25], [28], [29]); as well as observations of captive/farmed individuals (e.g., [30],[31]); and a small number of detailed observational studies conducted on wild populations (Table 1). However, no studies have previously been conducted using animal-borne imaging to directly assess the foraging behaviors of any crocodilian species [32].

**Table 1:** Published results regarding the feeding behaviors of crocodilians.

				Attack Frequency	Capture Success^c^	
Reference	Species	Hunting Mode^a^	Location^b^	Attacks/hour/ind.	Proportion	Habitat^e^
[28]	*Caiman crocodilus yacare*	Multiple	S	6.00	0.15	W
[35]	*C. crocodilus yacare*	Multiple	S	3.2†	0.05	W
[31]	*Gavialis gangeticus*	Sit-and-wait	S/SUB	4.1§	0.34	C
[36]	*C. crocodilus*	Surface snaps	S	0.075‡	0.07	W
–	–	Trapping	S	N/A	0.11	W
–	–	Fishing	S	N/A	0.29	W
–	–	Weir-fishing	S	N/A	0.44	W
–	–	Jumping	S	N/A	0.01	W

^a^ Multiple refers to combination of hunting behaviors.

^b^ Location in the water column where observations were made. S-surface or SUB-submerged.

^c^ Proportional success of prey-capture attempts, mean values are presented.

^e^ C-captive, W-wild.

Calculated from 35 attacks observed during 1.56 hours of observation of 7 wild caiman.

Calculated from 160 attacks observed during 4.85 hours of observation of 8 captive gharial.

Calculated as median value from minimum and maximum reported.

Due to cryptic foraging behaviors (i.e., nocturnal and submerged feeding), responsiveness to observer presence, and environmental constraints (i.e., water turbidity, vegetation density), few studies have succeeded in quantifying crocodilian foraging behaviors such as the frequency of prey-capture attempts (attacks) and the success rates of prey-capture attempts in natural settings (Table 1). Crocodilians, including American alligators, are considered nocturnal, opportunistic, generalist predators and are hypothesized to chiefly use sit-and-wait and/or ambush hunting at the water’s edge to capture prey [21], [23], [33], [34]. Additionally, a number of crocodilian species have been observed using specialized foraging behaviors such as cooperative feeding (for discussion see [29]) and the trapping and herding of fish (e.g., ‘cross-posture’-[35], ‘weir fishing’-[36]).

To gain a deeper understanding of alligators’ interactions within estuarine ecosystems, we used video data collected from Crittercam systems to answer the following questions: What is the frequency of prey-capture attempts? What factors contribute to variation in the frequency of prey-capture attempts? How successful are alligators at capturing prey? What factors influence the probability of prey-capture success? What are the diel activity patterns of alligators? How does time of day affect alligator activity patterns?

We compare our results to published estimates of prey-capture attempt frequency and prey-capture attempt success for other crocodilian species. Results from this work can be used in ecological models to estimate parameters regarding the strength and resulting impacts of American alligator’s trophic interactions within estuarine ecosystems. Our data demonstrate a potential for underestimation of prey-capture attempt and success rates of crocodilians when using limited direct observations and illustrate the importance of determining species- and system-specific ecological data to inform community- and ecosystem-scale impacts.

## Methods

### Ethics Statement

All animal care and use was approved by the University of Florida Institutional Animal Care and Use Committee (IACUC) (Protocols 201003798 and 201005071) and Kennedy Space Center IACUC (Protocol GRD-06-044). All field collections were performed under Florida Fish and Wildlife Conservation Commission Special Permit SPGS-10-44R and SPGS-10-43. Efforts were made to minimize discomfort and stress to animals while performing Crittercam attachment, and all study animals were released at the site of capture in a timely manner.

### Study Sites

**Figure 1:**
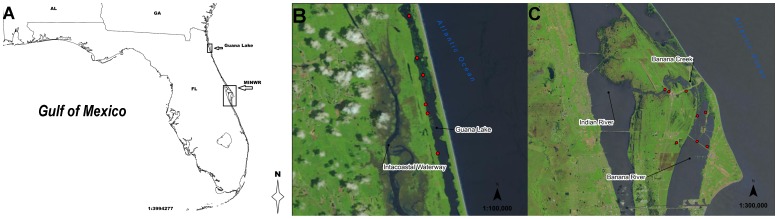
Study sites and capture locations. A) Map of southeastern United States, study areas are labeled by black boxes. B) Map of Guana Lake. C) Map of Merritt Island National Wildlife Refuge (MINWR). Red circles indicate capture and release locations of American alligators outfitted with Crittercam units. All image data was sourced from USGS National Map Viewer: http://viewer.nationalmap.gov/viewer/. Maps were created with Grass GIS analysis software (CC-BY-SA): http://grass.osgeo.org/.

Merritt Island National Wildlife Refuge (MINWR) comprises 56,655 hectares of freshwater wetland, upland scrub forest,as well as estuarine seagrass and mangrove habitats of Merritt Island (28.567145°N, 80.660039°W), a barrier island east of Cape Canaveral, Florida USA (Figure 1 A and B). MINWR was established in 1963 by the U.S. Fish and Wildlife Service, following development of the John F. Kennedy Space Center by the National Aeronautics and Space Administration. Although development has ensued since its’ establishment, large reaches of natural habitats remain intact and are protected from human use. The refuge supports a broad array of plants (over 1,000 species) as well as animal species (over 500 species), including one of Florida’s largest populations of West Indian manatee (*Trichechus manatus,* [37]). The micro-tidal estuaries of the Banana and Indian River Lagoons serve as vital foraging and nursery grounds for estuarine game fish, sharks and rays, migrating waterfowl, and wading birds [37]. Within estuarine habitats, multiple species of seagrass dominate fully submerged sub-tidal habitats, while intertidal shorelines are bordered by dense stands of mangrove, invasive Brazilian pepper (*Schinus terebinthifolius*), as well as various species of salt marsh grasses (*Spartina* ssp.), and shrubs. Throughout MINWR, freshwater marshes, retention ponds, dikes, and mosquito ditches establish a network of freshwater habitats utilized by alligators for reproductive activities such as mating and nesting, as well as freshwater and prey resources. In addition to consuming many freshwater and terrestrial prey species, alligators within MINWR regularly forage on prey resources from estuarine habitats (Rosenblatt et al, *in review*). Water salinity and temperature during Crittercam deployments (April 22 to May 6, 2010) were measured at a Banana Creek sampling station (28.589379°N, 80.659288°W) at approximately 0.8 meters depth (typical range for Banana Creek station: 0.8 to 1.2 meters) every 2 hours using a YSI 6920 EDS multiparameter sonde (YSI Incorporated, Yellow Springs, Ohio USA). Water salinity in estuarine habitats (i.e., Banana River and Banana Creek) was measure to be 16.45±0.9 ppt (mean ± SD). Using a single-way ANOVA, time of day (morning [0400–0900], day [0900–1800], evening [1800–2200], and night [2200–0400]) was found to significantly influence mean water temperature (F (3,176) = 19.23, *P*<0.001). Water temperatures (range = 22 to 30°C, 26.1±1.9°C [mean ± SD]) were highest during the day (26.9±1.7°C) and lowest during the evening (26.5±1.2°C), night (25.4±1.6°C), and morning (24.7±1.7°C), respectively. Post-hoc analysis (Tukey’s HSD test) indicated that water temperatures during the morning and nighttime periods were significantly less than day (morning-day *P*<0.001, night-day *P*<0.001) and evening (morning-evening *P*<0.001, evening-night *P* = 0.04) temperatures, but not significantly different from one another (*P* = 0.29). Water temperatures during the day and evening did not significantly differ (*P* = 0.69). Sunrise ranged from 06∶38 to 06∶47 EST, and sunset ranged from 19∶54 to 20∶00 EST throughout the duration of the study.

Guana Lake (30.086603°N, 81.3434877°W), a 930 hectare estuarine impoundment within Guana River Wildlife Management Area (4900 hectares) Ponte Vedra, Florida was created in 1957 by damming the upper reaches of the tidal Guana River ([38], Figure 1 A and C). The shallow (mean water depth ∼2 m) estuarine impoundment supports dense mats of submerged aquatic vegetation, chiefly Widgeon grass (*Ruppia maritima*) and filamentous algae. It is bordered by cattails (*Typha sp*.) in the northern reaches and Smooth cordgrass (*Spartina alterniflora*) and Black needle rush (*Juncus roemerianus*) in the southern reaches. Guana Lake, in addition to a robust population of American alligators, supports populations of small bait fish and minnows, game fish, wading birds, migratory waterfowl, small mammals, as well as a diverse assemblage of invertebrate taxa (e.g., shrimp, crabs, bivalves). A salinity gradient from north to south is maintained by fresh (0 ppt salinity) water inputs from rainwater runoff into the north and saline (24 to 34 ppt) water inputs from the tidal Guana River into the south. In drought conditions, water salinity can reach as high as 54 ppt and average ∼40 ppt throughout the impoundment (J.C. Nifong *personal observation*). During Crittercam deployments (April 27 to May 7, 2011) water salinity and temperature were measured at the North Dam sampling station (30.053160°N, 81.338750°W) at approximately 0.75 meters depth (typical range for depth at North Dam station: 0.53 to 0.86 meters) every 15 minutes using a YSI 6600 EDS (YSI Incorporated, Yellow Springs, Ohio USA) multiparameter sonde [39]. Mean ± SD water salinity was found to be 36.2±0.6 ppt and showed little variation throughout the study period. Using a single-way ANOVA, mean water temperature was found to be significantly affected by time of day (F (3, 236) = 36.52, *P*<0.001). Water temperatures (range = 21.2 to 28.5°C, mean ± SD = 25.2±1.9°C) were highest during the day (26.3±1.7°C) and lowest during the evening (25.7±1.2°C), night (24.6±1.5°C), and morning (23.7±1.6°C), respectively. Post-hoc analysis (Tukey’s HSD) revealed water temperatures during the morning and night hours were significantly less than temperatures during the day (morning-day *P*<0.001, night-day *P*<0.001) and evening (morning-evening *P*<0.001, night-evening *P* = 0.009). Temperatures in the morning were also significantly less than night (*P* = 0.009); while temperatures during day and evening did not significantly differ (*P* = 0.17). Sunrise ranged from 06∶38 to 06∶47 EST, and sunset ranged from 19∶59 to 20∶06 EST throughout the duration of the study.

### Alligator Capture and Crittercam Attachment/Detachment

From April 22 to May 6, 2010 and again from April 27 to May 7, 2011, a total of fifteen adult (>2m total length) *Alligator mississippiensis* (n = 9, MINWR in 2010; n = 6, Guana Lake in 2011, Figure 1 B and C, Table S1) were captured using standard crocodilian capture techniques (e.g., snatch hooks and rope snares). Once secured, individuals were subject to morphometric measurements (head length [HL], head width [HW], snout-to-vent length [SVL], total length [TL], and tail girth [TG]), blood sampling, scute tissue biopsies, and urine collection. Alligators were detained for approximately 20 minutes while Crittercam camera units were attached and all individuals were released immediately following sampling and attachment procedures.

**Figure 2:**
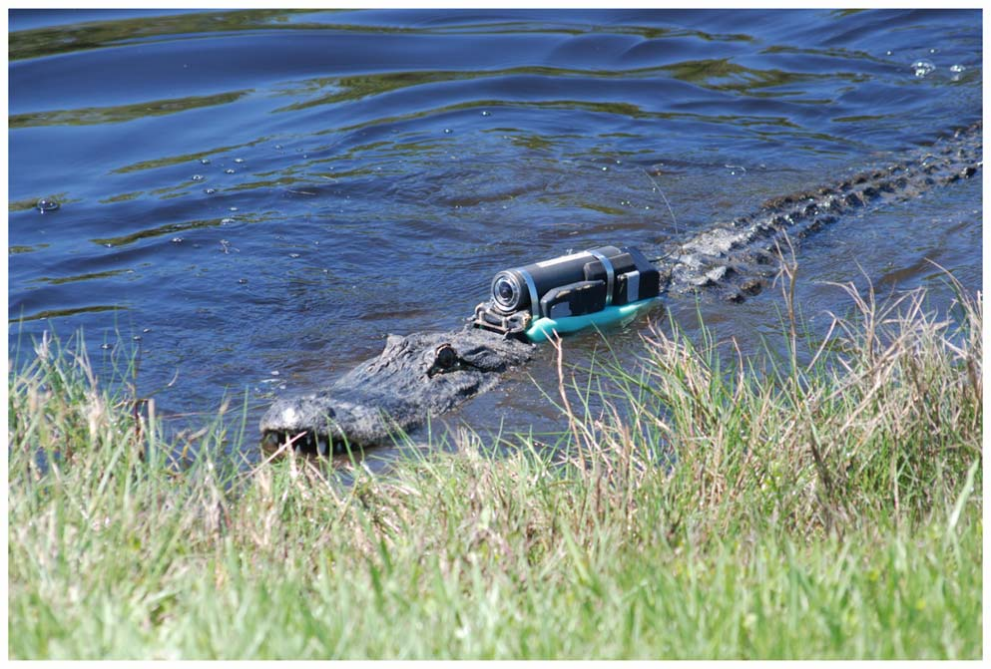
Crittercam unit attached to American alligator. Photograph of a 2.6 meter male American alligator (*Alligator mississippiensis*) with Crittercam unit attached. Reprinted with permission from J.C. Nifong.

Crittercam units were attached to alligators using a single-strap harness that automatically detached using a preprogrammed breakaway system (for detailed description of attachment and detachment methods see [32], Figure 2). Additionally, two failsafe breakaways were incorporated into the harness to ensure detachment in the case of system malfunction or the potential snagging of the Crittercam apparatus within the environment. The Crittercam and mount combination was designed to be marginally buoyant, to aid in recovery with minimal effect on the subjects.

### Data Collection

Each Crittercam unit housed a variety of onboard data sensors (e.g., acceleration, depth, temperature), a data storage device, 16 red LED lights, a battery pack, and one camera (either color or black and white video, both Hi 8 and 1080 P HD cameras were used). Due to the limited battery life and data storage capacity of the various Crittercam models used, a maximum of 6–8 hours of video data could be collected during any single deployment. Units were programmed for recording schedule, detachment time, and lighting (On/Off). Since time of day had previously been proposed to influence the frequency of prey-capture attempts as well as prey-capture success of crocodilians [28], [30], [36], we attempted to record video footage across all hours of the day. Cameras were set to record data for either 30 minute intervals or hour long intervals during a preprogrammed recording window. Depending on the length of the recording window and the type of recording schedule, Crittercam units were deployed for one to two days. To avoid the potential effects of stress related to capture, restraint, and release on alligator behavior, we attempted to set recording schedules to begin a minimum of 6 hours post-release [40].

Video data were downloaded from Crittercam units following detachment and recovery. Video data were then analyzed manually by trained technicians and checked for consistency. All videos were viewed and the activities of individual alligators cataloged as well as the duration of each activity, the habitat type, and time of day. Alligator activities encompassed a wide range of movements and interactions within the environment. To simplify our analysis of activity patterns, cataloged activities were grouped into six basic categories (foraging behaviors, sitting at surface, sitting submerged, swimming at the surface, swimming submerged, and on land). The on land behavioral category included both the activities of basking or sitting, and walking within the terrestrial environment including atop floating vegetation and emergent marsh. During two deployments alligators ventured into underwater dens; the occurrences of this behavior were lumped into the sitting submerged behavioral category. Foraging behaviors encompassed interactions with prey items such as prey-capture attempts, prey-captures, searching for prey (i.e., prey stalking and ‘head raking’-repetitive side to side head movements), and prey encounters. Crocodilians are inertial feeders that use a variety of head and body movements to capture, subdue, reposition, and swallow prey items [41]. Using this description of foraging behavior, prey-capture attempts were classified by an apparent head-strike/jaw-snap (attack) followed by no subsequent head movements such as surfacing, inertial bites (i.e., immobilization, crushing, repositioning), or swallowing ([42], Movie S1). Prey-capture successes were classified by an apparent head-strike/jaw-snap followed by subsequent inertial bites and swallowing ([42], Movie S2). If submerged at the time of prey capture, alligators often surfaced to immobilize, crush, reposition, and swallow captured prey.

### Statistical Procedures

We used generalized linear models (GLMs) to assess variation in the frequency of prey attacks and the probability of prey-capture success given an attack had occurred. We used Akaike Information Criterion (AIC) for model selection. We first selected the model with lowest AIC containing fixed effects from significant factors (*P*<0.05 on parameter estimates), then incorporated the random effect of ‘individual’ to see if our model deviance was improved. To estimate the frequency of prey attacks, we used a Poisson error distribution with a log link function to estimate the number of prey attacks per hour per alligator. Differences in sampling interval length were addressed using an offset within the Poisson GLM, taking into consideration 30 minute sampling intervals were half as long as one hour intervals. To estimate the probability of prey-capture success given an attack had occurred, we used a Binomial error distribution with logit link function. We assessed variation in the frequency of prey-capture attempts due to the factors of site/year, time of day, and individual. We assessed variation in the probability of prey-capture success due to the factors of site/year, time of day, position in water column prior to attack (submerged versus surface), hunting mode (active search versus sit-and-wait), habitat (in vegetation versus open water), and individual. All statistical analyses were performed in R 2.14.0, we used the glm () function in the package ‘stats’ to perform Poisson and logistic regressions [43]. Individual effects were included as random effects in our GLMs using the lmer () function in the package ‘lme4’ [44].

To evaluate alligator activity patterns, we first calculated the proportion of time spent performing six basic activities (foraging behaviors, sitting at surface, sitting submerged, swimming at surface, swimming submerged, and on land) across all viable video data (70.3 hours). We then analyzed the data separating by time of day and calculated the proportion of time spent performing activities during video recordings within four daytime intervals (Table S2). We performed a randomization procedure to determine if the time spent performing each basic activity was dependent on the time of day. We used the number of observations of each activity during each time of day and randomized the time spent performing that activity by drawing with replacement from the population-level distribution of activity durations (the elapsed time of each occurrence for each of the six behaviors). This process was repeated 10,000 times to create our null distribution. This null distribution represents how the population would behave if they spent the same amount of time performing activities regardless of the time of day. We assessed the significance of effects of time of day on activity patterns by calculating the two-sided *P*-values as the number of times the absolute difference between each randomization and the median of the null distribution was as extreme as the absolute difference between the observed proportions and the median of the null distribution, for each activity during each daytime interval, divided by the number of randomizations (10,000). All differences were considered significant at *P*<0.05.

## Results

### Deployments and Video Data

We outfitted 15 adult alligators (n = 9, MINWR in 2010; n = 6, Guana Lake in 2011, Figure 1) with Crittercam systems (4 females and 11 males, TL range 221 to 307 cm, mean ± SD = 262±25 cm, Figure 1 and 2, Table S1). No units were lost during deployments. In four of the deployments video recording ended early due to system malfunctions, early detachment, or camera lenses becoming obstructed by vegetation or substrate; this includes one deployment where no viable data was recovered. In total these losses resulted in ∼68.9% (70.3 hours) of the potential 102 hours of recordings being viable for use in our behavioral analyses (i.e., clear view of alligator’s head and surroundings). Alligators showed little signs of distress nor changes in behavior during deployments [32]. Due to the combined effect of a restricted field of view caused by the camera’s angle and position and the size of common prey species, we were unfortunately unable to identify species of prey during attacks or captures. However, stomach content analysis of individuals within the same season and locations found small prey species (e.g., small fish, crustaceans) to be the most frequently consumed prey items (Rosenblatt et al, *in review*).

### Frequency of Prey–capture Attempts

**Figure 3:**
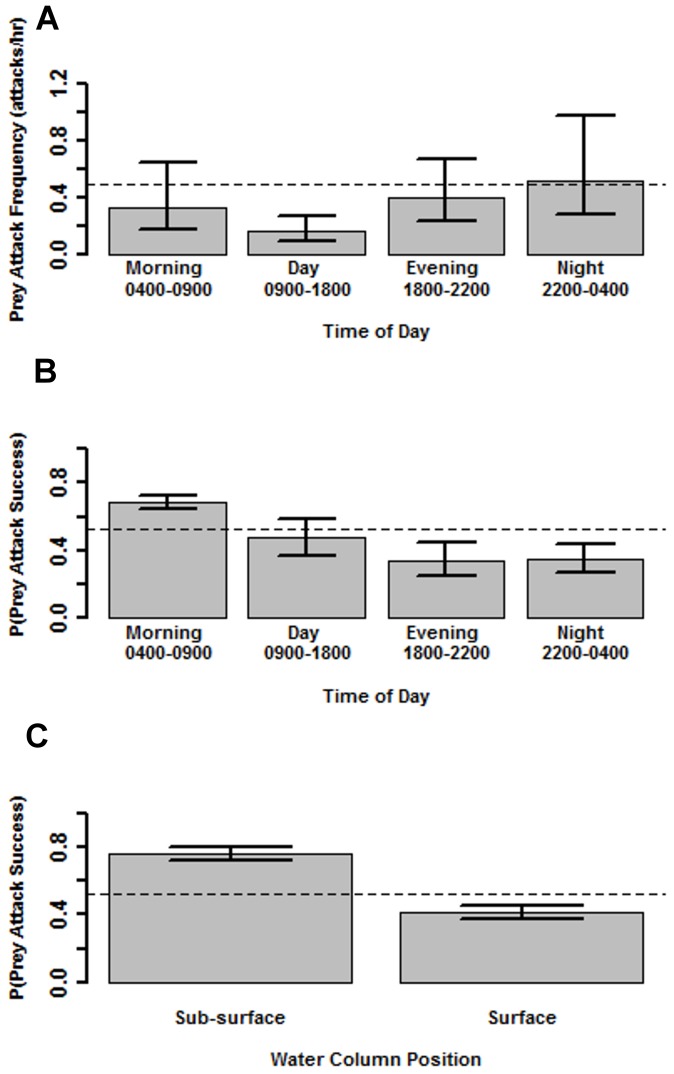
Alligator prey-attack frequency and prey-capture success. Estimated effects of A) time of day (Morning [0400–0900], Day [0900–1800], Evening [1800–2200], and Night [2200–0400]) on frequency of prey attacks from best fit Poisson GLM, B) time of day (Morning [0400–0900], Day [0900–1800], Evening [1800–2200], and Night [2200–0400]), and C) water column position prior to attack (submerged or surface) on the probability of prey-capture success. Bars are mean estimates and error bars Wald 95% Confidence Intervals. The dashed line is the overall mean frequency of prey attacks predicted by our null GLM.

We identified a total of 59 prey attacks (either attempts or captures) across a total of 120 sampling intervals. Nine of the 14 individuals (∼64%) performed at least one prey attack. The number of attacks ranged from 0 to 18 per hour, however only one individual performed more than 4 attacks in one hour. Since the maximum number of attacks (18 attacks in one hour) was potentially driven by different biological and statistical processes, we removed this data point from our analysis of prey-capture attempt frequency. This choice was supported by the fact that removing that data point reduced the variance due to the random effect of *individual* in our GLM by nearly 50%. Our final model for frequency of prey-attacks includes time of day as a fixed effect and *individual* as random effect (Table 2). Based on our null GLM, mean prey-capture attempt frequency was 0.49 prey-attacks/hour/alligator (Wald 95% CI = 0.42–0.58). Time of day significantly influenced the frequency of attacks, with attacks being most frequent in the nighttime hours and less frequent in the morning, evening, and day, respectively (Figure 3 A).

**Table 2:** AIC values for Poisson GLMs, predicting prey-capture attempt frequency.

		Delta AIC
Model	AIC	(Δ*_i_*)
Daytime+Individual	104.9	0.0
Daytime	179.0	74.1
Daytime+Site	179.6	74.7
Intercept (Null)	181.1	76.2

### Probability of Prey-capture Success

Of the 59 prey attacks recorded, 31 (∼53%) resulted in confirmed capture of prey. Since we were interested in the outcome of individual attacks, the “feeding frenzy” (18 attacks in one hour) was included in our analysis of prey-capture success. Our final model for probability of prey-capture success included the fixed effects of time of day and water column position (Table 3). The addition of *individual* as a random effect did not improve our model deviance. The overall mean probability of prey-capture success from our null GLM was 0.53 (Wald 95% CI = 0.46–0.59). Using our parameter estimates from our best fit model we independently assessed the variation in probability of prey-capture success due to time of day and water column position. The mean probability of prey-capture success was highest in the morning (mean P(capture success) = 0.68) and lowest during the day (0.47), night (0.34), and evening (0.34), respectively (Figure 3 B). An alligator’s position in the water column prior to prey attack, either submerged or above the water’s surface, greatly influenced the probability of prey-capture success. When foraging below the surface alligators were nearly twice as successful when compared to foraging at or above the water’s surface (Figure 3 C).

**Table 3:** AIC values for logistical GLMs, predicting the probability of prey-capture success.

		Delta AIC
Model	AIC	(Δ*_i_*)
Daytime+Water Position	77.4	0.0
Daytime+Water Position+Hunting Mode	77.5	0.1
Daytime+Water Position+Hunting Mode+Site+Habitat	78.4	1.0
Daytime+Water Position+Hunting Mode+Site	78.8	1.4
Daytime+Water Position+Hunting Mode+Individual	79.4	2.0
Null (Intercept)	83.6	6.2

### Alligator Diel Activity Patterns

**Figure 4:**
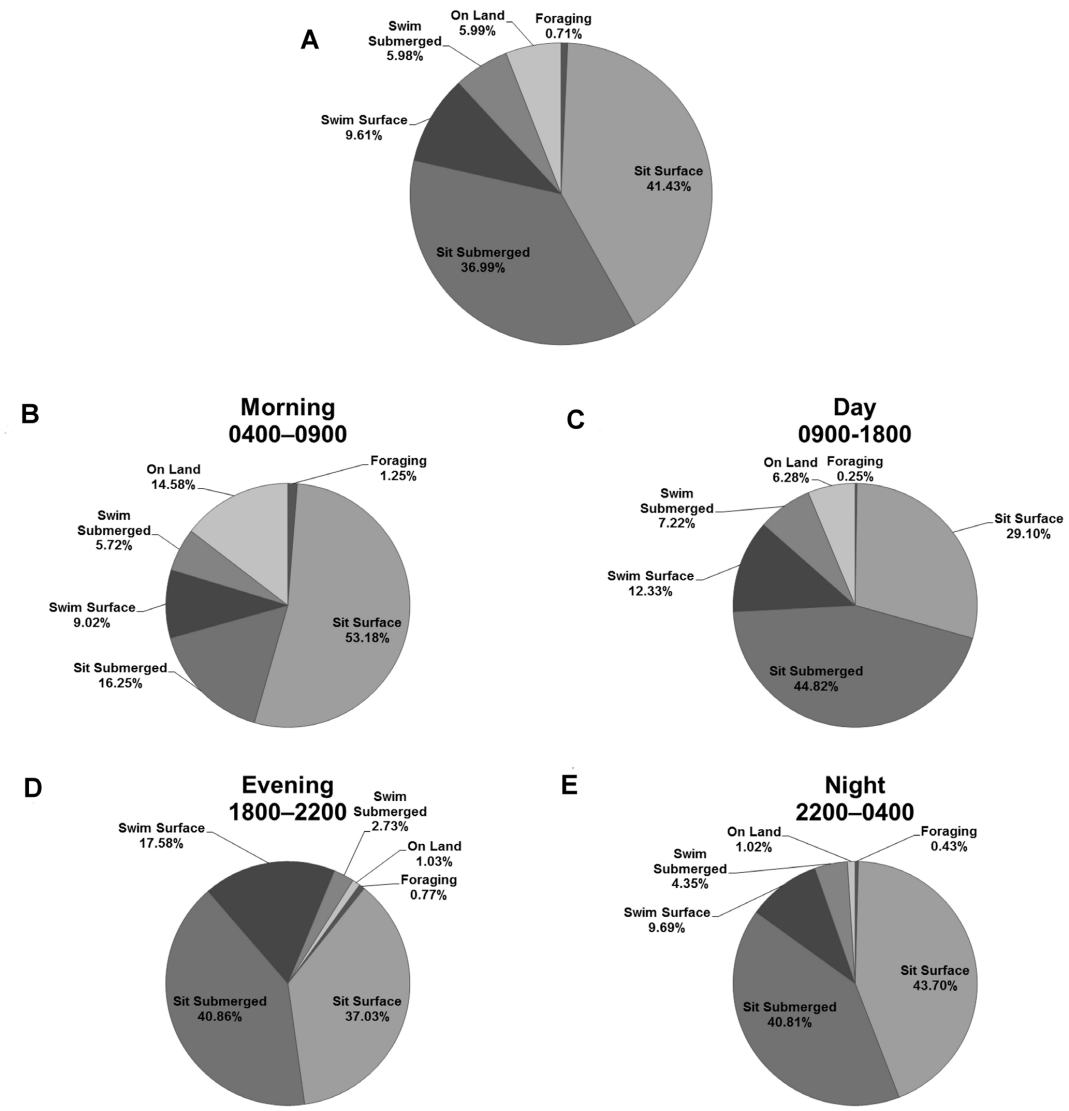
Alligator diel activity. Pie-charts of proportion of alligators spent time performing basic activities during A) all recordings, B) Morning (0400–0900), C) Day (0900–1800), D) Evening (1800–2200), and E) Night (2200–0400). Proportions are calculated as the sum of elapsed time performing an activity divided by the total time of video recordings during each time interval.

Overall, alligators primarily performed two basic activities for the greatest proportion of time we recorded, sitting at the water’s surface (41.4%) and sitting submerged (36.9%). Alligators spent less time swimming, either at the surface (9.6%) or submerged (5.9%); moreover, alligators spent the least amount of time on land (5.9%) and performing foraging behaviors (0.7%, Figure 4 A, Tables S1 and S2). Summing the proportion of time performing activities while submerged, alligators spent 42.8% of their time underwater. Interestingly the order (greatest to least) of proportional time spent performing basic activities changed very little throughout all hours of the day (Figure 4, Tables S2 and S3). Our randomization procedure only found the proportion of one activity for one daytime interval to be dependent on the time of day; alligators swam submerged for a significantly greater proportion of time (8.5%) during the day (Table 4). Although the time alligators spent performing activities were largely independent of time of day, a few patterns emerged from the data. In particular, alligators traveled onto land (10.1%) and remained at the water’s surface (53.6%) for the greatest periods of time during the morning (0400–0900 h). Additionally, alligators sat at the water’s surface during the day the least (29.4%) and were submerged the most compared to all other time intervals (54.4%).

**Table 4:** Two sided *P*-values from random permutation test for independence of proportion spent performing activities from time of day.

Daytime Interval	Foraging	SitSurface	SitSubmerged	SwimSurface	SwimSubmerged	On Land
Morning (0400–0900 h)	0.471	0.173	0.136	0.786	0.885	0.544
Day (0900–1800 h)	0.746	0.073	0.451	0.553	**0.025**	0.753
Evening (1800–2200 h)	0.467	0.686	0.430	0.210	0.192	0.549
Night (2200–0400 h)	0.497	0.091	0.308	0.871	0.284	0.183

**Bold** values indicate significant effect of time of day on the proportion of time spent performing an activity.

## Discussion

Large-bodied apex predators are important drivers of community structure and ecosystem function, through their cascading influence on lower trophic levels (for review see [45]). However, due to inherent difficulties ecologists face while studying many of these organisms, there remains a paucity of data regarding many aspects of their general biology, natural history, and ecology [4]. Here we used Crittercam, an animal-borne imaging system, to study the behavior of American alligators (*Alligator mississippiensis*) in two Florida estuaries, ecosystems which lack data regarding feeding behaviors and activity patterns of alligators [17], [46].

Through analysis of data recovered from Crittercam video recordings we found alligators to frequently attempt to capture prey (mean = 0.49 attacks/hour/individual) and moreover, that these attempts were often successful (mean rate of success = 53%). We estimate that on average, American alligators successfully consumed one prey item (or group of small prey species) every 4 hours in these locations during the time of this study. This estimate seems probable given the small size and high numbers of prey items in the stomach contents of individuals within these populations (Rosenblatt et al, *in review*).

The frequency of prey-attacks was dependent on the time of day (Figure 3). Consistent with their description as nocturnal or crepuscular predators, alligators attacked prey more frequently at night (2200–0400 h) and into the early morning hours (0400–0900 h). In the case of this study, the effect that time of day had on the frequency of prey-capture attempts was most likely driven by the combined effects of prey availability or accessibility and thermoregulatory behavior patterns. Small prey species (e.g., shrimp, top minnows, crabs, crayfish) are a major dietary component of alligators in these two estuarine populations (Rosenblatt et al, *in review*). These prey species often aggregate in dense schools at night to feed on nektonic prey resources (e.g., zooplankton) and seek shelter in thick vegetation mats during the day to escape predation and avoid heat stress (J.C. Nifong, *personal observation*, [47]). Alternatively, prey may have aggregated in response to the presence of the on-board lights used by Crittercam units during nighttime hours. Thus, increased prey attack rates during nighttime hours may have been partially driven by prey species’ attraction to light sources and a subsequent response by alligators to increase the number and frequency of prey encounters.

As ectothermic organisms, body temperature in crocodilians is maintained by behavior, chiefly basking (at water’s surface or on land) or changing body posture within the water column [48], [50]. When body temperatures are low, as in the early morning, many crocodilians use heat seeking behaviors such as traveling onto land to bask or positioning themselves horizontally at the water’s surface to warm via solar radiation; however, to maintain optimal body temperatures throughout the day crocodilians must use cold seeking behaviors as well, either by returning to aquatic habitats or altering their body position in the water column to exploit thermal gradients. Findings from our analysis of daily activity patterns suggest alligators perform heat seeking thermoregulatory behaviors such as traveling onto land and surface basking in response to rising temperatures beginning in the late morning hours (0700 to 0900 h), and then perform cold seeking behaviors throughout the day until the evening hours when temperatures begin to decrease. We also observed alligators engaging in night-time basking behavior or traveling onto land for a small percentage of recorded observations (1.2%, Figure 4, Table S1, S2, and S3). Thus, alligators may respond to increased prey abundance and availability as well as a concurrent, decreasing need to perform thermoregulatory behaviors beginning in the evening through the early morning hours by allocating more effort toward the acquisition of prey via increasing the frequency of prey-capture attempts.

To our knowledge, only two studies have quantitatively assessed the effect of time of day on the frequency of prey-capture attempts for any species of crocodilian. Schaller and Crawshaw (1982) used direct observations of *Caiman crocodilus yacare* in the Pantanal of Brazil to assess feeding behaviors in isolated ponds during the dry season (June to December). Prey-attack frequency of *C. crocodilus yacare* averaged 3.5 attacks/hour/caiman and was greatest in the morning between the hours of 0600 and 0900 h, and least in the day between 1000 and 1500 h. However, their observations took place from 0600 to 1600 h and included only those feeding behaviors observable above the water’s surface (i.e., weir fishing, surface snaps, trapping). Schaller and Crawshaw (1982) hypothesized satiation and social interactions were responsible for these patterns in *C. crocodilus yacare* feeding behaviors. Similar to the findings of Schaller and Crawshaw (1982), Thorbjarnarson (1993) found a bimodal pattern in the frequency of predatory attacks by *C. crocodilus* in the Venezuelan Llanos throughout the day. Prey-attack frequency ranged from 0 to 0.15 attacks/hour/caiman and was greatest in the late morning between the hours of 0600 to 0900 h and in the mid- to late-afternoon between 1300 to 1700 h. Thorbjarnarson (1993) also observed that the frequency of *C. crocodilus* fishing behaviors depended on season, and attributed this pattern in foraging behaviors to fluctuations in prey availability. The frequency of fishing behaviors by *C. crocodilus* reduced as fish prey became scarce during the later portions of the dry season. All observations were made during daylight hours (0600–1800 h), and only feeding behaviors visible above the water’s surface were considered for their analyses. In both of these studies researchers commented that while predatory events were commonly observed during night-time hours (1800–0600 h), no quantitative measures of feeding behaviors could be made due to low visibility and reaction of caiman to the presence of observers.

Temperature directly affects the frequency of prey-capture attempts by crocodilians through its effects on metabolic demands over both short (hours) and long (season) temporal scales [51], [52]. In the systems studied throughout the duration of Crittercam deployments, multiple time intervals were similar in temperature, but significantly differed in the frequency of prey-capture attempts (see Methods for discussion of temperature changes due to time of day). Water temperatures at all times of the day in both study locations were greater than the thermal minimum (20°C) observed for spontaneous feeding responses in caiman and alligators [48], [53]. In the short-term, temperature seems to affect the frequency of prey-capture attempts throughout the day indirectly via changing activity patterns due to behavioral responses of alligators (i.e., thermoregulatory behaviors-[49]) rather than having a direct effect via increasing metabolic demands. Our study was performed during mid-spring where water and air temperatures do not reach the maxima observed during summer and fall in Florida. Taking this into account, prey attack and consumption rates may vary seasonally due to changes in behavior or metabolic demands [54]. Comparative research should be performed over broader temporal scales to assess seasonal differences in alligator feeding behaviors.

Our estimated probability of prey-capture success for alligators is considerably higher than published estimates for crocodilians in other natural habitats (overall mean 1.2 to 52 times greater, depending on hunting mode/strategy, Table 1). In addition to the potential for differences in prey-capture success due to species, other published estimates evaluating hunting mode/strategy, habitats, and time of year (season) for other crocodilian species have not considered certain temporal and spatial components of crocodilian foraging behaviors. Previously reported estimates may be biased because those studies only estimated prey-capture success during the day (0600–1800 h), considered only those foraging attempts that occurred above the water’s surface, and noted the inability of researchers to track individuals performing repeated capture attempts (Table 1). Using animal-borne imaging we found only 22% of all foraging behaviors occurred between the hours of 0600 and 1800 h, corresponding to the times of day considered in previous studies. Additionally, 30% of all prey-capture attempts and successes we observed occurred while alligators were submerged and 36% occurred within thick vegetation; locations where direct observation of crocodilian behavior is difficult and highly limited.

Prey-capture success was significantly affected by both time of day and position in the water column prior to prey-capture attempts (Figure 3 B and C). The effects of time of day on the probability of capture success did not follow the same pattern as frequency of prey-capture attempts; alligators were most successful capturing prey during the morning and into the daytime hours and least successful at night and in the evening.

Schaller and Crawshaw (1982) assessed variation in the success of fishing behaviors of *C. crocodilus yacare* in Brazil. While observations were limited to daylight hours (0600–1600 h), the mean capture success rate of 15.9% varied throughout the day; similar to our findings for alligators, prey-capture success was highest in the morning and decreased from mid-day (1300 h) into the remaining hours of the afternoon observed in their study. Thorbjarnarson (1993) studied the success of fishing behaviors of *C. crocodilus* in the Llanos of Venezuela. He found prey-capture success to mirror the bimodal pattern found for frequency of prey-capture attempts; wherein capture success was highest in the late-morning (0800–1000 h) and mid-afternoon (1400–1600 h) and considerably lower during the remainder of observations. Patterns in prey-capture success of alligators due to time of day found in our study may partially be explained by prey availability and detectability. Conceivably, increased prey densities during the night would increase the frequency of both direct prey-capture attempts in response to visual cues and reactionary snaps in response to the stimulation of sensitive mechanoreceptors (integumentary sensory organs ISOs- [55],[56] or domed pressure sensors DPRs-[57]) on the head by schooling prey striking into the alligators’ jaw and head regions. Reactionary snaps are potentially less effective for prey capture, as opposed to visually motivated capture attempts and specialized feeding behaviors such as trapping (Table 1).

Position in the water column, either submerged or at the surface, prior to prey-capture attempts significantly affected the success of capture attempts (Figure 3 C). We found submerged alligators were nearly two times more successful than when capture attempts were made at the water’s surface (mean P(capture success) = 0.76 [submerged] and 0.41 [surface]). This study is the first to estimate the success of underwater prey-capture attempts for any crocodilian species in the wild. While no comparative data exists, we hypothesize the increased success of capture attempts when submerged may be driven by decreased maneuverability and evasion success of benthic prey species opposed to species occupying surface waters and nekton.

In this study an alligator’s hunting mode/strategy (either active search or sit-and-wait) did not significantly influence the probability of prey-capture success away from the mean (P(capture success) = 0.53); other studies have demonstrated considerable variation in the success of prey-capture attempts depending on the particular hunting technique employed (see Table 1). In particular, varieties of sit-and-wait hunting (e.g., floating, weir fishing) are reported as more successful than costly, specialized hunting techniques such as jumping (see [36] and [29] for description of crocodilian hunting techniques and specialized behaviors). In this study sit-and-wait predatory attacks accounted for 67% of prey-capture attempts by American alligators; this may have biased our results due to the lack of data regarding the success rate of capture attempts performed under active search strategies.

In addition to the effects of time of day, temperature, water column position, hunting mode/strategy, and individual differences; prey-attack frequency and capture success of alligators in this study were potentially influenced by water salinity. While alligators readily use hyper-saline habitats, they lack lingual salt glands maintained in many species of true crocodiles (family Crocodylidae) [58]; therefore, alligators must use behavioral mechanisms to regulate osmotic balance in hyper-saline environments. In terms of feeding behaviors, alligators may preferentially consume hypo-osmotic prey such as fish, may reduce their overall prey consumption, or the frequency of prey consumption while in hyper-saline habitats as compared to freshwater habitats. Further research comparing the feeding behaviors of estuarine and freshwater inhabiting populations will help to elucidate the potential differences in feeding behaviors employed by alligators in response to salinity. As such, caution should be taken when extrapolating findings from estuarine to freshwater populations.

Our analysis of prey-capture success included the data from the ‘feeding frenzy’ individual (i.e., 18 attacks in one sampling interval). We included this individual’s data in the analysis of prey-capture success because the outcome of each foraging attempt was the metric of interest as opposed to the number of prey-capture attempts per sampling interval. We are confident including this individual’s foraging behaviors in the data analysis of capture success did not bias the analysis since the inclusion of *individual* as a random effect in the binomial GLM for capture success did not improve the model deviance (Table 3). The inclusion of these data were further supported by the fact that when the ‘feeding frenzy’ data were removed from the analysis; the significance and order of effects from the factors of time of day and water column position prior to attack did not change (i.e., P(capture success) for morning>day>evening>night and P(capture success) for submerged>surface). This suggests that the ‘feeding frenzy’ data did not have a disproportionate effect on variation in capture success compared to other observations of prey-capture attempts.

The analysis of diel activity patterns yielded interesting insights into the behavior of American alligators in estuarine ecosystems. To our knowledge only one study has attempted to characterize fine-scale movement and/or activity patterns of alligators in estuarine habitats. Watanabe et al. (2013) studied the activity patterns of adult American alligators in the Banana River Lagoon (same population as this study) in fall of 2010 using multisensory data loggers (sensor recorded information on tri-axial acceleration, depth, temperature, and swim speed). While only four individuals were outfitted with data loggers and considerable variation existed between individuals, they found similar activity patterns to those observed while using Crittercam deployments in this study. Similar to our findings, alligators were active (swimming or walking), on average, roughly 20% of the time (Fig. 4, Table S2 and S3). Additionally, alligators were most active during the day (0600–1800 h), displaying short, inactive dives and periodic swimming behaviors. At night alligators were least active, alternating between sitting submerged and periodic surfacing. Watanabe et al. (2013) suggested behavioral patterns in the day were most likely linked to thermoregulation, while activity patterns during night were suggestive of foraging via sit-and-wait predation. Our findings using animal-borne imaging corroborate these assumptions; alligators foraged most frequently at night and during the early morning and performed thermoregulatory behaviors during late morning and daytime hours.

Accurate estimates of submergence rates and other factors that affect the detectability of crocodilians during night eye-shine counts (the most commonly used method for estimating crocodilian population trends) assists managers and researchers by refining the predictions of crocodilian population size through the incorporation of correction factors for count data[59], [60], [61]. We estimate alligators in this study were submerged, on average, for ∼48% of the time during the hours of 2000–0200 h, times when the majority of eye-shine surveys occur in the state of Florida (C. Carter, *personal communication.*). We found submergence rates of alligators, to remain relatively consistent (>40%) throughout all night time hours (2000–0700 h). Using data from radio telemetry units Bugbee (2008) found the submergence rates of alligators in the Florida Everglades to be affected by season, time of day, body size, body condition, sex, moon phase, water depth, and wind speed. Similar to this study, alligator submergence rates in the Everglades were highest during the day and lowest during the night. Although contrary to our findings, considerable variation existed during night-time hours, wherein submergence was highest during the second hour of night (post-sunset) and decreased later into the night (3 hours post-sunset) into the morning hours [61].

### Conclusions

Large-bodied, apex and top predators are important components of ecosystems due to the broad-scale impacts of their behavioral interactions with the prey and the surrounding environment [1], [62]. Understanding the foraging dynamics and behavior of these species can assist conservationists and wildlife managers by providing essential data regarding their interactions within ecosystems, an important task in the face of the rapid declines and losses of many apex and top predator species worldwide [1], [5], [63], [64]. Here, we used animal-borne imaging to estimate the frequency of prey-capture attempts, capture success rates, and diel activity patterns of American alligators in two Florida estuaries. We found time of day to greatly influence both the frequency of prey-capture attempts and the probability of capture success. Additionally, position in the water column influenced capture success, with alligators being nearly twice as successful when attempting prey captures while submerged. Our results are the first available estimates regarding nocturnal and submerged feeding behaviors for any crocodilian species in the wild, as very few studies have quantified crocodilian feeding behaviors through direct observation (Table 1).

These findings highlight the importance of determining species-specific information regarding trophic interactions and diel activity patterns as well as the utility of animal-borne imaging for the study of crocodilian ecology and behavior. Further research should be performed on alligator populations over broader spatial and temporal scales to assess variation in these behaviors over space and time. Moreover, we studied the feeding behavior and activity patterns of only one species out of the 23 recognized crocodilian species worldwide; further research should be performed to study the behavior of other crocodilian species for which little data exists and many of which are threatened or endangered [34]. Additionally, our study only included adult size classes due to the size constraints of our animal-borne imaging equipment; caution should be used when this data is applied to inform the interactions of sub-adult and juvenile size classes as their interactions and activity patterns may greatly differ from adult size classes. In conclusion, enabled by cutting-edge animal-borne imaging technology, this research is a major step forward in not only furthering the understanding of crocodilian interactions in natural ecosystems, but also in providing valuable insight regarding the feeding behavior of an ecologically important apex predator whose cryptic nature has historically hindered such research.

## Supporting Information

Table S1 **Crittercam deployment data.** Summary of all data from each individual American alligator outfitted with a Crittercam unit.
(XLSX)

Table S2 **Alligator diel activities.** Total time of usable video footage recorded, *n* the number of individuals the video data was recorded from, and the percent time observed performing basic activities.
(DOCX)

Table S3 **Summary table of alligator diel activity.** The cumulative (overall), minimum, and maximum percentage of time during daytime intervals alligators’ were observed performing basic activities.
(DOCX)

Movie S1 **Prey-capture attempt.** Video segment retrieved from Crittercam unit. Notice little subsequent movement following the initial jaw snap.
(MP4)

Movie S2 **Prey-capture success**. Video segment retrieved from Crittercam unit. Notice the subsequent jaw movements involved in repositioning of prey following capture.
(MP4)

## References

[1] Estes JA, Terborgh J, Brashares JS, Power ME, Berger J, et al (2011) Trophic downgrading of planet Earth. Science (New York, NY) 333: 301–306 10.1126/science.1205106 21764740

[2] Altmann J (1974) Observational Study of Behavior: Sampling Methods. Behaviour 49: 227–267 Available: http://www.jstor.org/stable/4533591 4597405 10.1163/156853974x00534

[3] Pauly D (2000) Ecopath, Ecosim, and Ecospace as tools for evaluating ecosystem impact of fisheries. ICES J Mar Sci 57: 697–706 10.1006/jmsc.2000.0726

[4] Williams TM, Estes JA, Doak DF, Springer AM (2004) Killer appetites: assessing the role of predators in ecological communities. Ecology 85: 3373–3384 10.1890/030696

[5] Heithaus MR, Frid A, Wirsing AJ, Worm B (2008) Predicting ecological consequences of marine top predator declines. Trends Ecol Evol 23: 202–210 10.1016/j.tree.2008.01.003 18308421

[6] Lazzaro X, Lacroix G, Gauzens B, Gignoux J, Legendre S (2009) Predator foraging behaviour drives food-web topological structure. J Anim Ecol 78: 1307–1317 10.1111/j.13652656.2009.01588.x 19619219

[7] Moll RJ, Millspaugh JJ, Beringer J, Sartwell J, He Z (2007) A new “view” of ecology and conservation through animal-borne video systems. Trends Ecol Evol 22: 660–668 10.1016/j.tree.2007.09.007 18006184

[8] Marshall GJ (1998) Crittercam: An animal-borne imaging and data logging system. Marine Technology Society Journal 32: 11–17.

[9] Dunson WA (1982) Salinity relations of crocodiles in Florida Bay. Copeia 1982: 374–385 Available: http://www.jstor.org/stable/1444618

[10] Mazzotti FJ, Dunson WA (1984) Adaptations of *Crocodylus acutus* and *Alligator* for life in saline water. Comp Biochem Physiol A Mol Integr Physio 79: 641–646 Available: http://linkinghub.elsevier.com/retrieve/pii/0300962984904626

[11] Mazzotti FJ, Best GR, Brandt L A, Cherkiss MS, Jeffery BM, et al (2009) Alligators and crocodiles as indicators for restoration of Everglades ecosystems. Ecol Indic 9: S137–S149 Available: http://linkinghub.elsevier.com/retrieve/pii/S1470160X08000770

[12] Craighead F (1968) The role of the American alligator in shaping plant communities and maintaining wildlife in the southern Everglades. The Florida Naturalist 21: 2–7.68–74.94.

[13] Kushlan J (1974) Observations on the role of the American alligator (*Alligator mississippiensis*) in the southern Florida wetlands. Copeia 1974: 993–996 Available: http://www.jstor.org/stable/10.2307/1442609

[14] Kushlan JA, Kushlan M (1980) Everglades alligator nests: nesting sites for marsh reptiles. Copeia 1980: 930–932 Available: http://www.jstor.org/stable/1444493

[15] Palmer ML, Mazzotti FJ (2004) Structure of Everglades Alligator Holes. Wetlands 24: 115–122 10.1672/0277-5212(2004)0240115:SOEAH2.0.CO2

[16] Subalusky AL, Fitzgerald LA, Smith LL (2009) Ontogenetic niche shifts in the American Alligator establish functional connectivity between aquatic systems. Biological Conservation 142: 1507–1514 Available: http://linkinghub.elsevier.com/retrieve/pii/S0006320709000950

[17] Rosenblatt AE, Heithaus MR (2011) Does variation in movement tactics and trophic interactions among American alligators create habitat linkages? J Anim Ecol 80: 786–798 10.1111/j.13652656.2011.01830.x 21418209

[18] Bondavalli C, Ulanowicz RE (1999) Unexpected Effects of Predators Upon Their Prey: The Case of the American Alligator. Ecosystems 2: 49–63 10.1007/s100219900057

[19] Keddy P A (2009) Thinking Big: A Conservation Vision for the Southeastern Coastal Plain of North America. Southeast Nat 8: 213–226 10.1656/058.008.0202

[20] Nifong J, Silliman B (2013) Impacts of a large-bodied, apex predator (*Alligator mississippiensis* Daudin 1801) on salt marsh food webs. J Exp Mar Bio Ecol 440: 185–191 Available: http://www.sciencedirect.com/science/article/pii/S0022098113000063

[21] Delany MF, Abercrombie C (1986) American alligator food habits in northcentral Florida. J Wildl Manage 50: 348–353 Available: http://www.jstor.org/stable/3801926

[22] Gabrey SW (2010) Demographic and geographic variation in food habits of American alligators (*Alligator mississippiensis*) in Louisiana. Herpetol Conserv Bio 5: 241–250 Available: http://www.herpconbio.org/Volume_5/Issue_2/Gabrey_2010.pdf

[23] McIlhenny EA (1935) The alligator’s life history. Boston, MA: The Christopher Publishing House. 117p.

[24] Jackson JF, Campbell HW, Campbell Jr KE (1974) The Feeding Habits of Crocodilians: Validity of the Evidence from Stomach Contents. J Herpetol 8: 378–381 Available: http://www.jstor.org/stable/1562912

[25] Pooley A (1989) Food and feeding habits. In: Ross CA, editor. Crocodiles and Alligators. Silverwater, Australia: Golden Press. 76–91.

[26] Platt S, Rainwater T (2006) Food habits, ontogenetic dietary partitioning and observations of foraging behaviour of Morelet’s crocodile (*Crocodylus moreletii*) in northern Belize. The Herpetological Journal 16: 281–290 Available: http://www.ingentaconnect.com/content/bhs/thj/2006/00000016/00000003/art00007

[27] Wallace KM, Leslie AJ (2008) Diet of the Nile Crocodile (*Crocodylus niloticus*) in the Okavango Delta, Botswana. J Herpetol 42: 361–368 10.1670/071071.1

[28] Schaller G, Crawshaw Jr PG (1982) Fishing behavior of Paraguayan caiman (Caiman crocodilus). Copeia 1982: 66–72 Available: http://www.jstor.org/stable/10.2307/1444269

[29] King FW, Thorbjarnarson J, Yamashita C (1998) Cooperative Feeding, A Misinterpreted and Under-Reported Behavior of Crocodilians. Available: http://www.flmnh.ufl.edu/herpetology/herpbiology/bartram.htm.

[30] Whitefield AK, Blaber SJM (1979) Predation on Striped Mullet (*Mulgil cephalus*) by *Crocodylus niloticus* at St. Lucia, South Africa. Copeia 1979: 266–269 Available: http://www.jstor.org/stable/1443412

[31] Thorbjarnarson J (1990) Notes on the feeding behavior of the gharial (*Gavialis gangeticus*) under semi-natural conditions. J Herpetol: 17–19. Available: http://www.jstor.org/stable/1564301

[32] Nifong JC, Lowers RH, Silliman BR, Abernathy K, Marshall G (2013) Attachment and Deployment of Remote Video/Audio Recording Devices (Crittercams ) on wild American alligators (*Alligator mississippiensis* ). Herpetol Rev 44 (2): 243–247.

[33] Wolfe J, Bradshaw DK, Chabreck RH (1987) Alligator feeding habits: new data and a review. Northeast Gulf Science 9: 1–8.

[34] Martin S (2008) Global diversity of crocodiles (Crocodilia, Reptilia) in freshwater. Hydrobiologia 595: 587–591 10.1007/s10750-007-9030-4

[35] Olmos F, Sazima I (1990) A fishing tactic in floating Paraguayan caiman: the cross-posture. Copeia 1990: 875–877 Available: http://www.jstor.org/stable/10.2307/1446458

[36] Thorbjarnarson J (1993) Fishing behavior of spectacled caiman in the Venezuelan Llanos. Copeia 1993: 1166–1171 Available: http://www.jstor.org/stable/10.2307/1447104

[37] U. S. Fish and Wildlife Service (2013) Merritt Island National Wildlife Refuge. Available: http://www.fws.gov/merrittisland/.

[38] Frazel D (2009) Site profile of the Guana Tolomato Matanzas National Estuarine Research Reserve. Ponte Vedra, FL. Available: http://www.gtmnerr.org.

[39] National Oceanic and Atmospheric Administration, Office of Ocean and Coastal Resource Management NERRSMP (2004) Centralized Data Management Office. Available: http://cdmo.baruch.sc.edu.

[40] Watanabe Y, Reyier E, Lowers R, Imhoff J, Papastamatiou Y (2013) Behavior of American alligators monitored by multi-sensor data loggers. Aquat Biol 18: 1–8 doi:.3354/ab00489

[41] Gans C (1969) Comments on inertial feeding. Copeia 1969: 855–857 Available: http://www.jstor.org/stable/10.2307/1441816

[42] Cleuren J, de Vree F (1992) Kinematics of the jaw and hyolingual apparatus during feeding in Caiman crocodilus. J Morphol 212: 141–154 10.1002/jmor.1052120205 29865591

[43] R Core Development Team (2011) R: A Language and Environment for Statistical Computing. Available: http://www.r-project.org/.

[44] Bates D, Maechler M, Bolker B (2011) lme4: Linear mixed-effects models using S4 classes. Available: http://cran.r-project.org/package=lme4.

[45] Terborgh J, Estes JA, editors (2010) Trophic Cascades: Predators, Prey, and the Changing Dynamics of Nature. Washington, D.C.: Island Press. 453 p.

[46] Nifong JC, Rosenblatt AE, Johnson NA, Barichivich W, Silliman BR, et al (2012) American Alligator Digestion Rate of Blue Crabs and Its Implications for Stomach Contents Analysis. Copeia 2012: 419–423 10.1643/CE-11-177

[47] Rozas L, Minello T (1998) Nekton use of salt marsh, seagrass, and nonvegetated habitats in a south Texas (USA) estuary. Bull. Mar. Sci. 63: 481–501 Available: http://www.ingentaconnect.com/content/umrsmas/bullmar/1998/00000063/00000003/art00003

[48] Lang JW (1979) Thermophilic response of the American alligator and the American crocodile to feeding. Copeia 1979: 48–59 Available: http://www.jstor.org/stable/1443728

[49] Smith EN (1979) Behavioral and physiological thermoregulation of crocodilians. Am Zool 19: 239–247 Available: http://www.jstor.org/stable/3882433

[50] Fish FE, Cosgrove LA (1987) Behavioral thermoregulation of small American alligators in water: Postural changes in relation to the thermal environment. Copeia 1987: 804–807 Available: http://www.jstor.org/stable/1445682

[51] Coulson RA, Hernandez T (1964) Biochemistry of the alligator: A study of metabolism in slow motion. Baton RougeLA: Louisiana State University Press. 138 p.

[52] Olegario C, Diefenbach C (1988) Thermal and feeding relations of *Caiman latirostris* . Comp Biochem Physiol A Mol Integr Physiol 89: 149–155.

[53] Diefenbach CO (1975) Gastric function in *Caiman crocodilus* (Crocodylia: Reptilia). I. Rate of gastric digestion and gastric motility as a function of temperature. Comp Biochem Physiol A Mol Integr Physiol 51: 259–265 Available: http://www.ncbi.nlm.nih.gov/pubmed/237653 10.1016/0300-9629(75)90369-2 237653

[54] Lewis LY, Gatten RE (1985) Aerobic metabolism of American alligators, Alligator mississippiensis, under standard conditions and during voluntary activity. Comp Biochem Physiol A Mol Integr Physiol 80: 441–447 Available: http://www.ncbi.nlm.nih.gov/pubmed/2858324 10.1016/0300-9629(85)90065-9 2858324

[55] Brazaitis P (1987) Identification of crocodilian skins and products. In: Webb GJ, Manolis SC, Whitehead PJ, editors. Wildlife Management: Crocodiles and Alligators. Chipping Norton, NSW: Surrey Beatty & Sons. 373–386.

[56] Leitch DB, Catania KC (2012) Structure, innervation and response properties of integumentary sensory organs in crocodilians. J Exp Biol 215: 4217–4230 10.1242/jeb.076836 23136155 PMC4074209

[57] Soares D (2002) An ancient sensory organ in crocodilians. Nature 417: 241–242 10.1038/417241a 12015589

[58] Taplin LE, Grigg GC, Harlow P, Ellis TM, Dunson WA (1982) Lingual salt glands in Crocodylus acutus and C. johnstoni and their absence from Alligator mississippiensis and Caiman crocodilus. J Comp Physiol B 149 (1): 43–47.

[59] Magnusson WE (1982) Techniques of surveying for crocodilians. Proceedings of the 5th Annual Working Meeting of the Crocodile Specialist Group of the Species Survival Commission of IUCN-The World Conservation Union. Gland, Switzerland. 389–403.

[60] Woodward AR, Rice KG, Linda SB (1996) Estimating Sighting Proportions of American Alligators During Night-light and Aerial Helicopter Surveys. Proceedings of the Annual Conference of Southeastern Association of Fish and Wildlife Agencies. Vol. 32601: 509–519.

[61] Bugbee CD (2008) Emergence dynamics of American alligators (*Alligator mississippiensis*) in Arthur R. Marshall Loxahatchess National Wildlife Refuge: Life history and application of statewide alligator surveys. Master's Thesis. University of Florida. 115 pp.

[62] Rosenblatt AE, Heithaus MR, Mather ME, Matich P, Nifong JC, et al (2013) The roles of large top predators in coastal ecosystems: New insights from Long Term Ecological Research. Oceanography 26 (3): 156–167 10.5670/oceanog.2013.59

[63] Baum JK, Worm B (2009) Cascading top-down effects of changing oceanic predator abundances. J Anim Ecol 78: 699–714 10.1111/j.13652656.2009.01531.x 19298616

[64] Myers RA, Worm B (2003) Rapid worldwide depletion of predatory fish communities. Nature 423: 280–283 10.1038/nature01610 12748640

