# Supplementary material for: Animal-Borne Imaging Reveals Novel Insights into the Foraging Behaviors and Diel Activity of a Large-Bodied Apex Predator, the American Alligator (Alligator mississippiensis)
Source: PLoS One. 2014 Jan 15;9(1):e83953. doi: 10.1371/journal.pone.0083953 (PMC3893291; doi:10.1371/journal.pone.0083953)
Supplement: Table S2 — Alligator diel activities. Total time of usable video footage recorded, n the number of individuals the video data was recorded from, and the percent time observed performing basic activities. (DOCX) [file pone.0083953.s002.docx]

| **Daytimes** | **Total Time Recorded (hr)** | **n** | **Foraging** | **Sit Surface** | **Sit Submerged** | **Swim Surface** | **Swim Submerged** | **On Land** |
| --- | --- | --- | --- | --- | --- | --- | --- | --- |
| All | 70.3 | 14 | 0.7% | 41.4% | 36.9% | 9.6% | 6.0% | 5.3% |
| Morning | 15.7 | 6 | 1.4% | 53.6% | 18.3% | 10.2% | 6.5% | 10.1% |
| Day | 23.9 | 10 | 0.3% | 29.4% | 44.2% | 10.3% | **8.5%** | 7.4% |
| Evening | 16.2 | 7 | 0.9% | 42.8% | 44.7% | 7.4% | 3.1% | 1.2% |
| Night | 14.4 | 7 | 0.5% | 46.7% | 36.7% | 10.4% | 4.7% | 1.2% |

**Bold** indicates significant reliance of the proportion of time spent performing an activity on the time of day
