# Supplementary material for: Animal-Borne Imaging Reveals Novel Insights into the Foraging Behaviors and Diel Activity of a Large-Bodied Apex Predator, the American Alligator (Alligator mississippiensis)
Source: PLoS One. 2014 Jan 15;9(1):e83953. doi: 10.1371/journal.pone.0083953 (PMC3893291; doi:10.1371/journal.pone.0083953)
Supplement: Table S3 — Summary table of alligator diel activity. The cumulative (overall), minimum, and maximum percentage of time during daytime intervals alligators’ were observed performing basic activities. (DOCX) [file pone.0083953.s003.docx]

| **Activity** | **Overall** | **Minimum** | **Maximum** |
| --- | --- | --- | --- |
| Foraging | 0.7% | 0.3%† | 1.4%* |
| Sit Surface | 41.4% | 29.4%† | 53.6%* |
| Sit Submerged | 36.9% | 18.3%* | 44.7%‡ |
| Swim Surface | 9.6% | 7.4%‡ | 10.4%§ |
| Swim Submerged | 6.0% | 3.1%‡ | **8.5%†** |
| On Land | 5.3% | 1.2%§ | 10.1%* |

**Bold** indicates significant reliance of the proportion of time spent performing an activity on the time of day.

*Morning (0400–0900 hours)

†Day (0900–1800 hours)

‡Evening (1800–2200 hours)

§Night (2200–0400 hours)
